# Incidence of new coding for dry eye and ocular infection in open-angle glaucoma and ocular hypertension patients treated with prostaglandin analogs: Retrospective analysis of three medical/pharmacy claims databases

**DOI:** 10.1186/1471-2415-11-14

**Published:** 2011-06-14

**Authors:** Gail F Schwartz, Sameer Kotak, Jack Mardekian, Joel M Fain

**Affiliations:** 1 Glaucoma Consultants, Greater Baltimore Medical Center; Wilmer Eye Institute, Johns Hopkins University, Baltimore, Maryland, USA; 2 Pfizer Inc, New York, New York, USA

## Abstract

**Background:**

To investigate the clinical relevance of two different preservative formulations, we compared 1-year incidence rates of additional coding of dry eye, ocular infection, or ocular surface disease (either dry eye or ocular infection) in open-angle glaucoma and ocular hypertension patients newly treated with latanoprost with benzalkonium chloride (BAK) or with travoprost-Z with SofZia^®^.

**Methods:**

This was a retrospective study of three U.S.-based patient-centric medical/pharmacy claims databases (MedStat, PharMetrics, i3-Ingenix). Patients were eligible if they filled a prescription for latanoprost or travoprost-Z between October 2006 and Q2 2008 (prescription date = index date) AND were continuously enrolled 6 months prior through 12 months after the index date AND had any open-angle glaucoma or ocular hypertension diagnosis within 90 days prior to the index date AND did not have an ocular surface disease diagnosis during the 180 days prior to the index date AND if they had not had a prescription for the index agent in the 180 days prior to the index date. Time to incidence of new coding for ocular surface disease in the first year post-index was estimated with a composite endpoint: diagnosis of dry eye or ocular infection by ICD-9-CM or Current Procedural Terminology code OR by prescription for cyclosporine ophthalmic emulsion or ocular antibiotics.

**Results:**

In all, 15,933 patients were treated with latanoprost and 7670 with travoprost-Z. Over 1 year, 4.3% of latanoprost and 4.5% of travoprost-Z patients were identified with dry eye (p = 0.28), and 10.9% and 11.1%, respectively, were identified with an ocular infection (p = 0.79). The 1-year incidence of new coding for ocular surface disease also was similar across treatments (13.9% vs 14.3%, respectively; p = 0.48).

**Conclusions:**

The retrospective analysis of three large prescription databases revealed that open-angle glaucoma and ocular hypertension patients newly treated with latanoprost preserved with BAK or travoprost-Z preserved with SofZia did not differ statistically in rates of dry eye, ocular infection, or ocular surface disease (either dry eye or ocular infection) during the first year post-index. Claims-based analyses are limited by nonrandomization and the inability to account for over-the-counter use or samples.

## Background

Ocular surface disease is an umbrella term that encompasses both dry eye disease (e.g., aqueous deficient and evaporative) and non-dry eye disease (e.g., lid-related diseases, allergic conjunctivitis, infective and noninfective keratitis) [1]. A question remains as to whether commercial preservatives used in ocular hypotensive eye drops administered by patients with open-angle glaucoma or ocular hypertension affect the occurrence of ocular surface disease, in particular the occurrence of dry eye and/or ocular infection, when the clinical evidence from randomized, controlled trials suggests there is no clear link [2-4].

All preservatives have the potential to cause corneal and conjunctival changes, including possibly dry eye. The prevalence of clinically diagnosed dry eye among glaucoma patients in general and among those treated with ocular hypotensive agents containing benzalkonium chloride (BAK) remains unclear [5,6], although relatively few patients included in pivotal trials of bimatoprost, latanoprost, and travoprost, all of which contain BAK, reported dry eye (1% to 4% for latanoprost and travoprost; 3% to 10% for bimatoprost) [2-4]. Moreover, removing BAK from a prostaglandin analog or using an alternative preservative may not reduce the occurrence of dry eye. A double-masked, randomized, parallel group trial [7] that compared the safety and tolerability of travoprost with BAK versus that of travoprost-Z with SofZia^®^, an ionic buffered system composed of boric acid, propylene glycol, sorbitol, and zinc chloride, found no statistically or clinically significant between-treatment differences with regard to ocular adverse events or tolerability; the prevalence of dry eye was 1.7% with travoprost-Z versus 2.0% with travoprost [8].

With regard to infection, preservatives protect against potentially dangerous bacterial and fungal organisms that may be introduced inadvertently into multiple-dose containers during eye drop instillation, especially with larger size bottles (e.g., 5 mL, 7.5 mL) [9-11]. The bactericidal and fungicidal activity of preservatives helps ensure ocular safety, an especially important consideration given the increase in bacterial resistance to antibiotic treatment of ocular infections [12,13] and the dramatic rise in the incidence of fungal keratitis [14-17]. Although these reported outbreaks of fungal keratitis occurred in contact lens wearers and we are not aware of published data suggesting that different preservatives may be associated with outbreaks with chronic use of ocular hypotensive medications, the dramatic rise in the incidence of the condition highlights the importance of using preservatives that comply with stringent standards of antimicrobial activity in multidose containers.

To investigate the clinical relevance of two different preservative formulations, we compared 1-year incidence rates of additional coding of dry eye, ocular infection, and ocular surface disease, defined as either dry eye or ocular infection, in open-angle glaucoma and ocular hypertension patients newly treated with latanoprost with BAK or travoprost-Z with SofZia.

## Methods

This was a retrospective analysis of information from three U.S.-based patient-centric medical and pharmacy claims databases: MedStat, PharMetrics, and i3-Ingenix. Together, these databases contain information from managed care organizations consisting of over 123.5 million patients. The data represent a systematic sample of commercial health plan information obtained from managed care plans throughout the U.S. It is paid claims data, which by definition is information collected by the medical plans from medical service providers to facilitate the adjudication and payment of health insurance benefits on behalf of the plan's enrolled members. The databases were deidentified in accordance with Health Insurance Portability and Accountability Act requirements prior to being made available for analysis in this study; use of these data in health research is exempt from institutional review board review.

Condition identifiers are provided in Table 1. Open-angle glaucoma and ocular hypertension were defined by ICD-9-CM codes, while dry eye, ocular infection, and the ocular surface disease composite endpoint reflecting either dry eye or ocular infection were defined by ICD-9-CM codes, *Current Procedural Terminology *[CPT] codes and/or prescriptions.

**Table 1:** Identifiers of Conditions

Condition	Identifier	Heading
Open-angle glaucoma	*ICD-9-CM:*	Open-angle glaucoma
	365.11	

Ocular hypertension	*ICD-9-CM:*	Ocular hypertension
	365.04	

Dry eye	Prescription*	Restasis (cyclosporine ophthalmic emulsion)
	*ICD-9-CM:*	
	370.20	
	370.21	Superficial keratitis, unspecified
	370.33	Punctate keratitis
	370.8^†^	Keratoconjunctivitis Sicca not specified as Sjogren's
	370.9^†^	Other forms of keratitis
	372.20^†^	Unspecified keratitis
	372.39^†^	Blepharoconjunctivitis
	375.15	Other conjunctivitis
	710.2	Tear film insufficiency unspecified
	*CPT:*	Sicca syndrome
	68760	
	68761	Closure of lacrimal punctum; by thermocauterization ligation or laser surgery
		Closure of lacrimal punctum by plug

Infection	Prescription*	Bacitracin (bacitracin); Bleph-10; Cetamide; Chibroxin; Ciloxan (ciprofloxacin); Erythromycin; Levaquin (levofloxacin); Neomycin; Neosporin; Oculflox (ofloxacin); Ocusulf-10; Sodium Sulamyd; Sulf-10; Tobradex; Vigamox (moxifloxacin); Zymar
	*ICD-9-CM:*	
	370.00	
	370.8^†^	Corneal ulcer unspecified
	370.9^†^	Other forms of keratitis
	372.00	Unspecified keratitis
	372.03	Acute conjunctivitis
	372.20^†^	Other mucupurulent conjunctivitis
	372.30	Blepharoconjunctivitis
	372.39^†^	Conjunctivitis unspecified
		Other conjunctivitis

Ocular Surface Disease Composite Endpoint	Any identifier of dry eye or ocular infection	

*Generic drug name in parenthesis where applicable.

^†^*ICD-9-CM *code applicable for both dry eye and infection.

CPT = Current Procedural Terminology; ICD-9-CM = International Classification of Diseases, Ninth Revision, Clinical Modification.

The derivation of the analysis population is summarized in Table 2. The patient's fill date for latanoprost or travoprost-Z was considered to be the index date. Patients were eligible for inclusion if the index date was between October 2006, when travoprost-Z was introduced in the U.S., and the second quarter of 2008 AND if they were continuously enrolled 6 months prior through 12 months after the index date AND if they had any open-angle glaucoma or ocular hypertension diagnosis within 90 days prior to the index date AND if they did not have a diagnosis of ocular surface disease during the 180 days prior to the index date AND if they had not had a prescription for the index agent in the 180 days prior to the index date. Of the more than 264,000 potentially eligible patients across the three databases, 23,603 were included in the analysis population.

**Table 2:** Derivation of analysis population

	Database			
	MedStat	PharMetrics	i3-Ingenix	Total
All patients in the database	41,105,604	>60,000,000	22,689,623	>123,795,227
And filled prescription for latanoprost or travoprost-Z 10/2006 - Q2/2008	152,961	69,298	42,424	264,683
And continuous enrollment 6 months prior through 12 months after index date	84,871	35,447	23,091	143,409
And any open-angle glaucoma or ocular hypertension* diagnosis within 90 days prior to index date	31,760	14,713	9107	55,580
And no ocular surface disease* during 180 days prior to index date	28,711	12,833	7597	49,141
And no prescription for the index agent in 180 days prior to index date	** 15,154**	** 4151**	** 4298**	** 23,603**

*Defined in Table 1.

Index date = date of initial prescription for latanoprost or travoprost-Z; Q2 = second quarter.

Frequencies of patients in the latanoprost and travoprost-Z groups diagnosed with dry eye, ocular infection, or ocular surface disease during 1 year of follow-up were tabulated separately and compared using Cochran-Mantel-Haenszel tests stratified by claims database and by indicator of condition. Kaplan-Meier and Cox proportional hazards survival analyses of time to occurrence of dry eye, ocular infection, or ocular surface disease were performed. The statistical significance of between-group differences in time to occurrence of each endpoint was assessed using the Cox proportional hazards model that included treatment, age, gender, Charlson Comorbidity Index (which is used routinely to characterize the health status of patients in administrative databases [18]), geographic region, diagnosis, and claims database as predictors. P-values for age and Charlson Comorbidity Index are from an analysis of variance model that included treatment, database, and treatment by database interaction. P-values for diagnosis, gender, and geographic region are from Cochran-Mantel-Haenszel tests stratified by database.

## Results

The analysis set included 15,933 patients prescribed latanoprost and 7670 patients prescribed travoprost-Z. Patients prescribed the two prostaglandins were similar with regard to gender and geographic region; a larger proportion of latanoprost-treated patients was diagnosed with ocular hypertension, and, on average, those in the latanoprost group were older and had a higher score on the Charlson Comorbidity Index (Table 3).

**Table 3:** Patient characteristics

Characteristic	Latanoprost N = 15,933	Travoprost-Z N = 7670	p-value
Diagnosis, n (%)			<0.0001
Open-angle glaucoma	13,918 (87.4)	6837 (89.1)	
Ocular hypertension	2015 (12.6)	833 (10.9)	
Sex, n (%)*			0.55
Female	8413 (52.8)	4008 (52.3)	
Male	7519 (47.2)	3662 (47.7)	
Age, years: Mean ± SD	67.4 ± 13.3	66.3 ± 13.0	<0.0001
Charlson Comorbidity Index:			
Mean ± SD	0.74 ± 1.25	0.67 ± 1.15	<0.0001
Geographic region^†^			0.17
Northeast	2914 (18.3)	1071 (14.0)	
Midwest	5004 (31.4)	2440 (31.9)	
South	5270 (33.1)	2756 (36.0)	
West	2728 (17.1)	1387 (18.1)	

*Missing data for 1 latanoprost patient.

^†^Missing data for 17 latanoprost and 16 travoprost-Z patients.

SD = standard deviation.

Over 1 year, 4.3% of latanoprost and 4.5% of travoprost-Z patients were identified with dry eye (p = 0.28), and 10.9% and 11.1%, respectively, were identified with an ocular infection (p = 0.79; Table 4). The 1-year incidence of new coding for ocular surface disease also was similar across treatments (13.9% vs 14.3%, respectively; p = 0.48). Among the 3,306 ocular surface disease events, there was no statistically significant between-treatment difference in proportions of patients developing the condition when it was indicated by diagnosis, CPT code, or prescription (Table 5).

**Table 4:** One-year incidence of new coding for dry eye ocular infection, or both dry eye and ocular infection,* n (%)

Index drug	Total patients	Dry eye	Ocular infection	Both
Latanoprost	15,933	600 (3.8)	1670 (10.5)	60 (0.4)
Travoprost-Z	7670	298 (3.9)	821 (10.7)	23 (0.3)
p-value	-	0.48	0.86	0.31

*Categories are not mutually exclusive. See Table 1 for definitions of dry eye and ocular infection.

**Table 5:** One-year incidence of new coding for ocular surface disease (OSD) by indicator of condition, n (%)

			OSD indicated by*					
			
	Total		Diagnosis (ICD-9-CM)		CPT Code		Prescription	
	
Index drug	No OSD	OSD	No OSD	OSD	No OSD	OSD	No OSD	OSD
Latanoprost	13,723 (86.1)	2210 (14.0)	15,127 (94.9)	806 (5.1)	15,849 (99.5)	84 (0.5)	14,275 (89.6)	1658 (10.4)
Travoprost-Z	6574 (85.6)	1096 (14.3)	7279 (94.9)	391 (5.1)	7616 (99.3)	54 (0.7)	6832 (89.1)	838 (10.9)
p-value	0.48		0.66		0.13		0.42	

*Categories are not mutually exclusive. See Table 1 for definition of ocular surface disease composite endpoint.

CPT = Current Procedural Terminology; ICD-9-CM = International Classification of Diseases, Ninth Revision, Clinical Modification.

**Figure 1:**
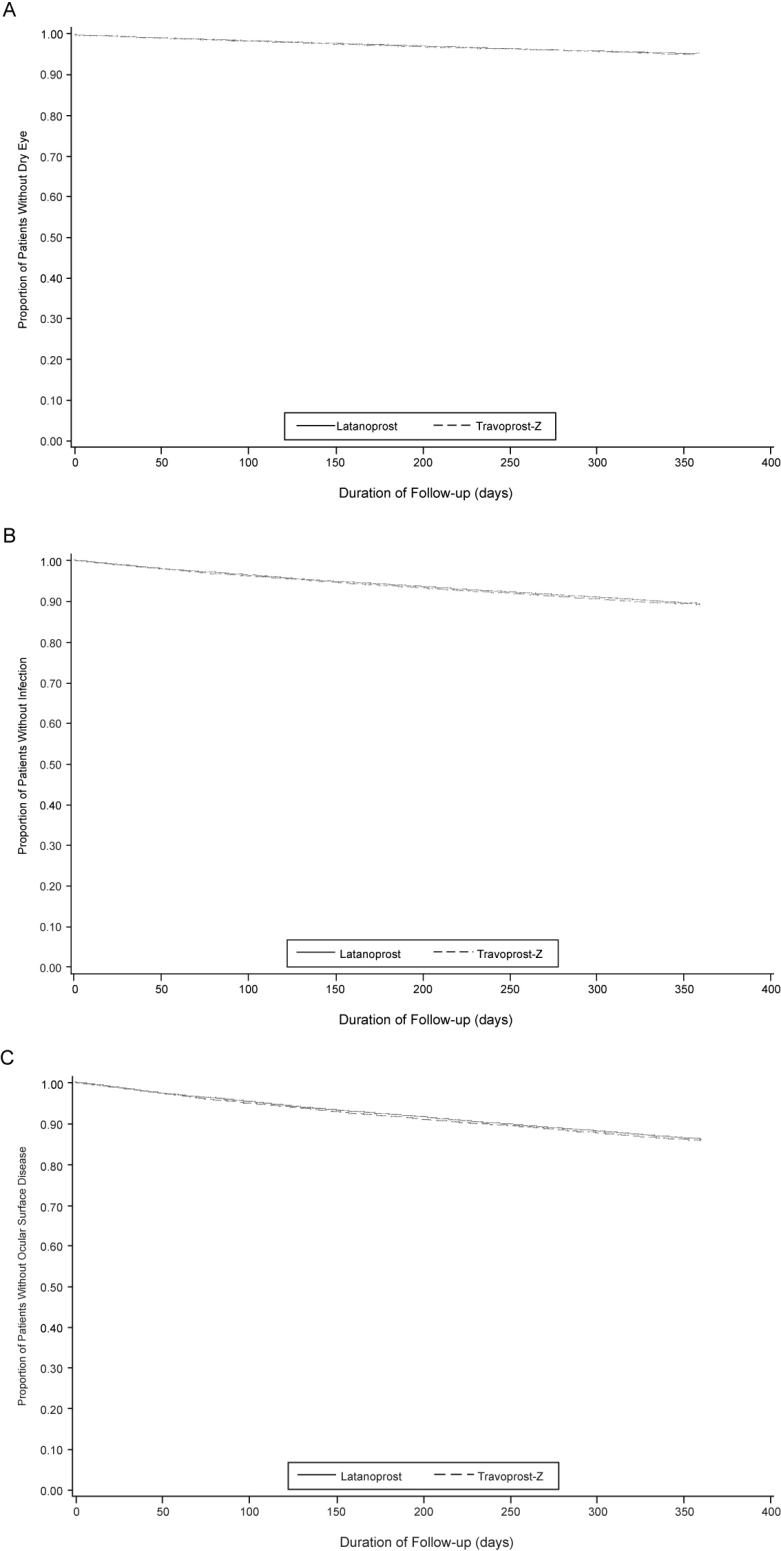
Days to dry eye (A), infection (B), and ocular surface disease composite endpoint (C) by treatment group

**Figure 2:**
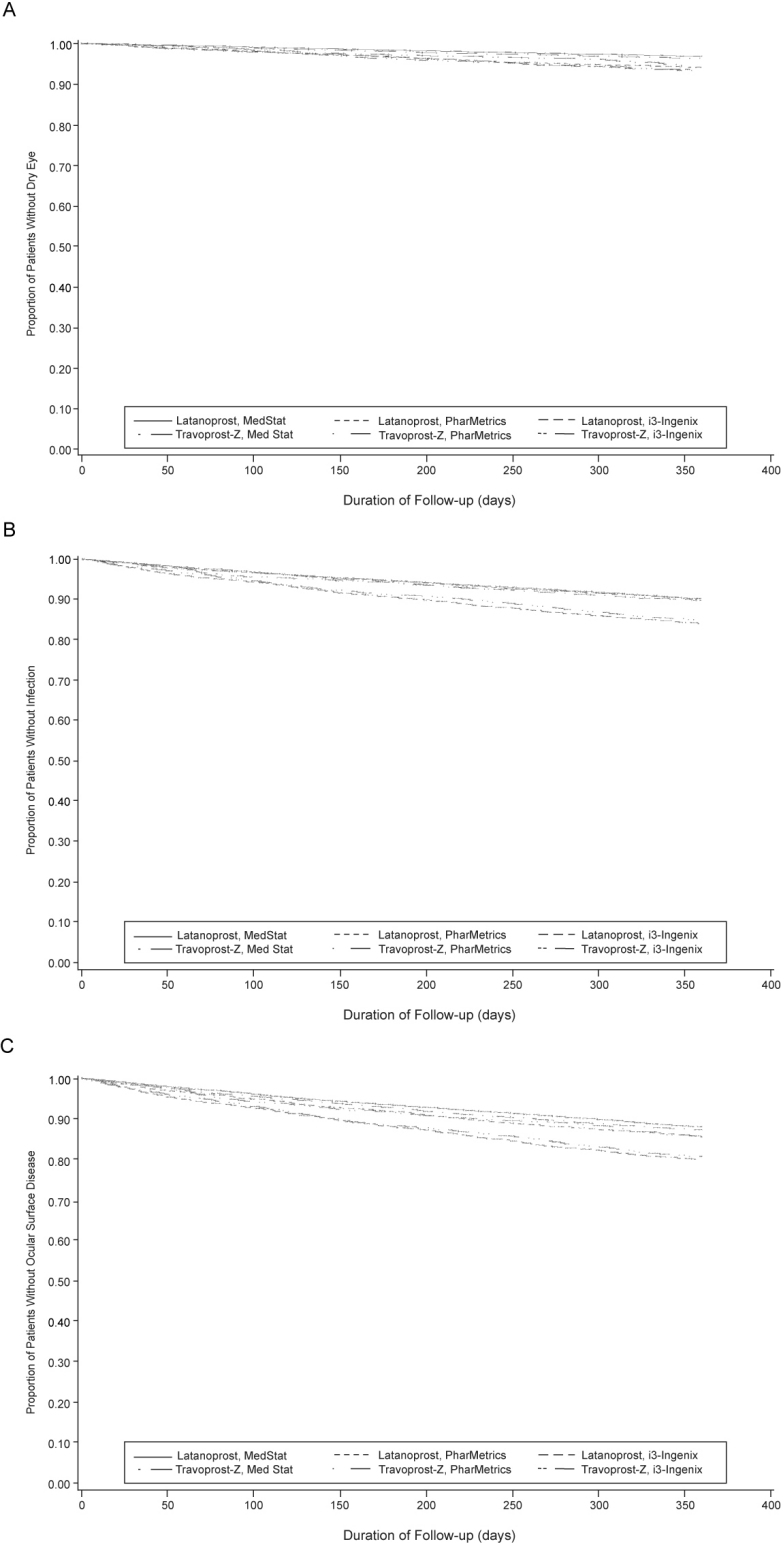
Days to dry eye (A), infection (B), and ocular surface disease composite endpoint (C) by database

**Figure 3:**
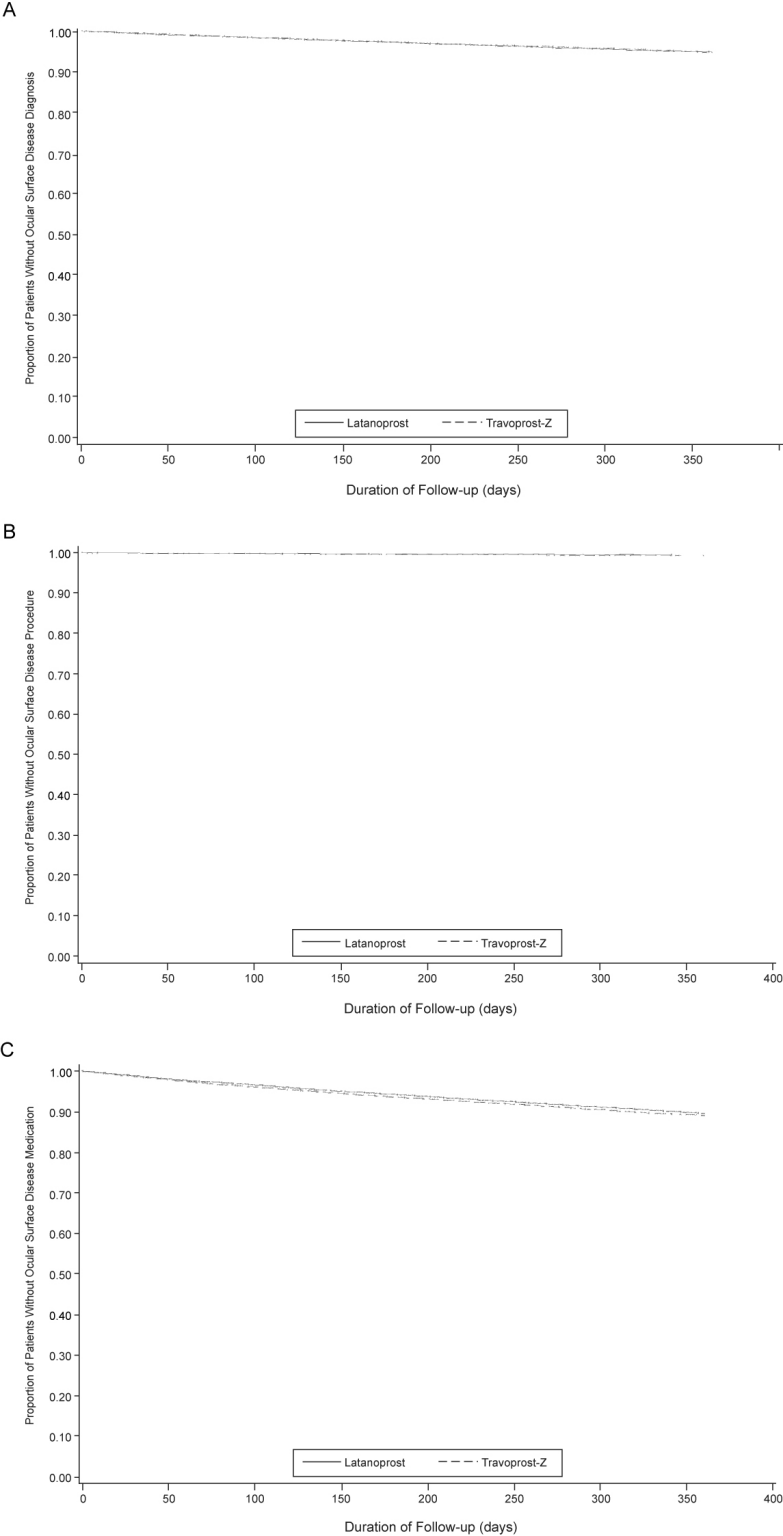
Days to ocular surface disease indicated by diagnosis (A), procedures (CPT code) (B), or prescription (C) by treatment group

Kaplan-Meier survival curves for time to dry eye, infection, and ocular surface disease were nearly identical for latanoprost and travoprost-Z overall (Figure 1 A, B, and 1C, respectively) and when stratified by database (Figure 2 A, B, and 2C, respectively. Curves also were virtually identical when ocular surface disease was indicated by diagnosis, CPT code, or prescription (Figure 3A, B, and 3C, respectively). Mean time to an ocular surface disease event among patients with events was approximately 5 months in both groups (latanoprost: 164.0 ± 104.8 days; travoprost-Z: 160.4 ± 104.3 days).

## Discussion

When choosing an ocular hypotensive agent for patients with open-angle glaucoma or ocular hypertension, physicians should consider the efficacy of candidate medications, their tolerability and side effect profiles, as well as any concomitant ocular and systemic conditions the patient may have [19]. With the advent of new preservative formulations, it also is essential that clinicians familiarize themselves with the body of evidence regarding the long-term safety and efficacy of the preservatives contained in ophthalmic solutions. Our analysis of three large prescription databases found that open-angle glaucoma and ocular hypertension patients newly treated with latanoprost containing BAK were not significantly more likely to develop dry eye, ocular infection, or ocular surface disease (either dry eye or ocular infection) as evidenced by additional coding for these disorders during the first year of treatment than were patients newly treated with travoprost-Z containing SofZia.

BAK, which has been used as a preservative for more than 50 years, is among the few preservatives that meet the rigorous criteria required by both the United States Pharmacopoeia (USP) and the European Pharmacopoeia [20,21]. SofZia, approved by the Food and Drug Administration in 2006, also meets USP standards, but little is published about its pharmacokinetics and pharmacodynamics [22]. There is contradicting evidence concerning the relationship of BAK and the development of ocular surface disorders. Dose-dependent, BAK-induced epithelial cellular damage has been found in studies of cultured corneal [23] and conjunctival cells [24] as well as in studies of cats and rabbits [25-28], but these studies may not accurately reflect ocular surface conditions in humans. Others [29-33] have shown that the levels of BAK contained in ophthalmic solutions are unlikely to cause clinically important negative corneal effects. Determining the relationship between BAK and dry eye in glaucoma patients is made more problematic by the fact that the incidence of both conditions increases with age [34,35], although history of glaucoma has not been found to be an independent risk factor for dry eye [36].

While preservative-free ocular hypotensive eye drops generally have been associated with fewer side effects [37-42] and better stability of the tear film [40,43] than drops containing a preservative, preservatives are added to ophthalmic preparations that are instilled multiple times in order to control microbial growth and to prevent the consequences associated with the use of contaminated solutions [21,44]. Serious infections can result from the instillation of contaminated eye preparations. Pathogenic contamination of eye drops is a significant risk factor for several complications, including infectious keratitis [21], although we know of no published data suggesting that different preservatives may be associated with outbreaks of this condition with chronic use of glaucoma medications.

The importance of determining the clinical impact of alternative preservatives in ocular hypotensive eye drops reflects the need to reduce the risk of ocular infection while limiting side-effects such as dry eye that might reduce patient adherence and persistence. Although we found no statistically significant difference in the 1-year incidence of ocular surface disease in more than 23,000 patients treated with either latanoprost or travoprost-Z, other studies [45-48] have yielded disparate results, at least in part due to differences in methodologies and measures, and, in some cases [46-48], to small samples and low power to detect differences if they existed. For example, a multicenter, investigator-masked, parallel-group study [47] of patients with glaucoma or ocular hypertension who had been treated with latanoprost monotherapy for at least 4 weeks were randomized to receive monotherapy with bimatoprost with BAK (n = 35), latanoprost with BAK (n = 38), or travoprost with SofZia (n = 33). Across 3 months of follow-up, no significant between-treatment differences were noted in conjunctival hyperemia scores, corneal staining, or tear breakup time. A randomized, double-blind study [48] compared tolerability in 33 patients treated with latanoprost in one eye and travoprost-Z in the other. Eyes were assessed by one examiner every 3 to 4 weeks for 3 months, and patients rated the extent of differences between eyes in ocular dryness/irritation at the beginning and at the end of the study. In this small sample, eyes treated with travoprost-Z had significantly more corneal staining (p = 0.025) and showed a trend toward more dryness and irritation symptoms than those treated with latanoprost (p = 0.095). A prospective, 8-week, unmasked, single-center study [46] of 40 eyes of 20 patients with low baseline tear break-up times who were switched from latanoprost to travoprost-Z found a significant increase (p < 0.001) in mean tear break-up time and a significant decrease (p < 0.001) in the mean Ocular Surface Disease Index score. The authors noted that the study focused on patients with low initial tear break-up times, limiting generalizability to the entire population of patients with dry eye; the lack of blinding, the absence of a comparison group, and the potential for regression to the mean also were limitations. A prospective, open-label, 3-month study [45] of 691 patients switched to travoprost-Z from latanoprost or bimatoprost due to tolerability issues found clinically and statistically significant improvement in ocular surface disease symptoms. The study was limited by the facts that open-label, switch designs do not control for expectations of improvement on the part of both patients and physicians, and by its short follow-up time frame.

As with all research, the present study has both strengths and limitations. Primary strengths include the large sample size. However, this was a retrospective analysis in which patients were not randomized, a basic limitation of all claims-based analyses. Patient use of over-the-counter products and samples as well as off-label use of topical corticosteroids to treat dry eye were not reflected in the database. The proportion of patients recommended to use artificial tears cannot be determined using a database analysis. Because this was a claims-based analysis rather than a clinical trial, we could not directly measure dry eye syndrome but instead measured the addition of a second code for one of many ocular surface diseases; we cannot estimate the frequency with which physicians omitted a relevant second code or patients added an artificial tear supplement without the knowledge of the physician. Identifying dry eye using CPT and ICD-9-CM codes may select for more severe cases of the condition. It is possible that the populations of patients using the two compounds could have been different since travoprost-Z has been specifically marketed and may have been chosen in some individuals who might already have mild, non-coded ocular surface disease prior to 180 days or who had a history of ocular surface symptoms that were not specifically coded for in the study. Not accounted for were any diagnosis that was made >180 days prior to the index date and that might not have been coded for as the subject developed glaucoma or the physician only coded for one, not two diseases which is quite common. In addition, although a glaucoma specialist (GFS) reviewed and approved the codes used to identify the clinical endpoints evaluated, other specialists might argue for the inclusion of other identifiers and/or the exclusion of some used herein; to our knowledge, there is no widely accepted set of identifiers for the general conditions of dry eye, ocular infection, or ocular surface disease.

## Conclusion

Results of the present retrospective analysis of three large prescription databases suggest that open-angle glaucoma and ocular hypotensive patients newly treated with latanoprost with BAK or with travoprost-Z containing SofZia do not differ statistically in rates of coding for dry eye, ocular infection, or ocular surface disease (either dry eye or ocular infection), during the first year post-index. Prospective, randomized, adequately powered comparisons of similarly effective ocular hypotensive agents with different preservatives are needed to more definitively answer the question of the clinical relevance of alternative preservative formulations.

## Competing interests

Dr. Schwartz was a paid consultant to Pfizer Inc in connection with the design and conduct of the study, including development of the manuscript. Dr. Fain, Dr. Mardekian, and Mr. Kotak are employees of Pfizer Inc. The study was supported by Pfizer Inc, New York, New York, USA.

## Authors' contributions

All authors participated in the study design, interpretation of data, and critical revision of the manuscript for important intellectual content, and all read and approved the final manuscript. SK provided study supervision, and JM facilitated acquisition and analysis of data from databases.

## Pre-publication history

The pre-publication history for this paper can be accessed here:

http://www.biomedcentral.com/1471-2415/11/14/prepub
